# How to age positively: perspectives of older adults with a low socioeconomic status using participant-driven photo-elicitation interviews

**DOI:** 10.1007/s10433-026-00909-w

**Published:** 2026-01-31

**Authors:** Feline Platzer, Martine M. Goedendorp, Nardi Steverink, Jiska Vorstman, Mathieu de Greef

**Affiliations:** 1 Department Gezondheid en Leven, Municipal Health Service, Assen, The Netherlands; 2 Department of Psychology, Faculty of Behavioural and Social Sciences, University of Groningen, Groningen, The Netherlands; 3 Department of Sociology, Faculty of Behavioural and Social Sciences, University of Groningen, Groningen, The Netherlands; 4 Department of Health Psychology, University Medical Center Groningen, University of Groningen, Groningen, The Netherlands; 5 Niped - Personal Health Check, Amsterdam, The Netherlands; 6 Department of Health Studies, Hanze University of Applied Science, Groningen, The Netherlands; 7 Department of Human Movement Sciences, University Medical Center Groningen, University of Groningen, Groningen, The Netherlands

**Keywords:** Photo-elicitation, Photo-voice, Low socioeconomic status, Older adults, Health positive ageing

## Abstract

Investigating health and ageing with older adults with low socioeconomic status (SES) is challenging, because they seem to have trouble completing questionnaires containing abstract concepts such as health and ageing. Visual tools may be more suitable alternatives. Therefore, we had the following research questions: What are the perspectives of low-SES older adults on positive ageing? How do low-SES older adults experience the participant-driven photo-elicitation method? For this study, 21 Dutch participants (mean age 75 years) gathered a maximum of 10 photographs about positive ageing. Most participants collected photographs digitally, and two participants needed assistance. Two weeks later, they were interviewed and asked to reflect on their photographs and share their experience with this photo-voice method. Results showed that most photographed themes were Social Health (spending time with children and grandchildren), Activities, Nature and Animals (pets), and Foods and Drinks. Additionally, Mental Health (feeling happy and accepting losses) and Positive Ageing (focusing on the bright side of life) were also discussed. All participants said that the method was pleasant and interesting. For some it made it easier to talk about positive ageing. Perceptions concerning positive ageing involved a combination of spending time with loved ones, activities, nature, pets, eating and drinking, and also accepting losses and enjoying life. An integrated approach could be directed at these perceptions in positive health programmes for older adults with a low SES.

## Introduction

Health disparities between people with a low and high socioeconomic status (SES), often measured as a combination of occupation, education and income (Shavers 2007), are striking. In the Netherlands, the life expectancy of people with a lower education has been almost five years shorter than that of people with a higher education over the last decades, and their perceived health is about 20% lower (van der Lucht et al. 2024). This gap widens with age (Prus 2004).

Studies have shown that most studies on health outcomes and the perspectives of older adults with low SES concerning ageing have been based on interviews and questionnaires (Kornadt et al. 2021; Steptoe and Zaninotto 2020), and also more ‘creative’ techniques, such as prioritising tasks (Flinterman et al. 2019). Although these studies provide valuable insights, the investigation of health and ageing with this target group is regarded as challenging (Flinterman et al. 2019). For instance, older adults with low SES are difficult to reach for research purposes, and they seem to have trouble completing questionnaires containing abstract concepts (e.g. health), due to literacy problems and cognitive apprehension (Federman et al. 2009; Flinterman et al. 2019).

The use of visual methods in research might offer an opportunity to gain more insight into the health experiences and perceptions of older adults with low SES (Platzer et al. 2021a; 2021b). First mentioned by the researcher and photographer John Collier (1957), methods involving the use of photographs in research are known as photo-elicitation techniques. A distinction can be made between researcher-driven photo-elicitation techniques, in which researchers collect the photographs, and participant-driven photo-elicitation techniques (or ‘photo-voice’), in which participants take the photographs (Catalani and Minkler 2010; Wang and Burris 1997).

Several studies have used participant-driven photo-elicitation with older adults to investigate their perspectives on physical health, mental health, the age-friendliness of the community, age and identity, chronic pain and intergenerational housing (Baker and Wang 2006; Killion and Wang 2000; Kohon and Carder 2014; Novek et al. 2012; Shell 2014; Sims-Gould et al. 2010). Researchers have reported that the photo-voice technique is useful for gaining insight into the personal experiences of older adults, as well as the challenges they face and the social issues that affect their lives (Novek et al. 2012; Mysyuk and Huisman 2020). The technique has also been reported to raise consciousness among participants with regard to their perceptions and the communities in which they live (Wang and Redwood-Jones 2001).

Although previous studies have explored the subjective and objective health of older adults with low SES, to our knowledge, none has used photo-voice to explore perceptions on positive ageing. Positive ageing refers to an individual’s ageing process, including several positive conditions such as physical vitality, optimal cognitive functioning, having or building meaningful relationships and the opportunity to participate in the society (Fernandez-Ballesteros 2011; World Health Organization 2020). Our aim was therefore to use participant-driven photo-elicitation to explore the perceptions of older adults with low SES concerning positive ageing. Because this method has not previously been used with this target group, we also describe its feasibility and experiences of participants with its use. Our research questions are as follows; what are perspectives of low-SES older adults on positive ageing? How do low-SES older adults experience the participant-driven photo-elicitation method used in the study?

## Method

### Preparation of the study

The aim of this study was to explore participants’ perceptions on positive ageing using an explorative qualitative research design. Participants were asked to gather photographs about positive ageing and, subsequently, to discuss these photographs during an individual interview. We pre-tested the study before conducting the interviews. The Ethics Committee of the Faculty of Behavioural and Social Sciences of the University of Groningen reviewed and approved this study (PSY-2021-S-0538). Participants gave active written informed consent to participate in the study and written informed consent to use their photographs in this study.

### Testing the method

From their own networks, two of the authors (FP and JV) recruited two older women (84 and 65 years of age) with low SES and tested the interview with them. Both participants received a presentation about the study and took photographs during a two-week period. Thereafter, participants were interviewed about the photographs according to an interview format. When discussing the photographs, the researchers noticed that one test participant had gathered only photographs of herself. When the interviewer asked whether she had any other ideas about positive ageing that she had not photographed, she explained the benefits of reading and technology (e.g. laptops and tablets) for positive ageing. She had not taken any photographs relating to these topics, as she would not have been in the photograph herself. The second test participant brought 14 photographs to the test interview. To limit the interview time to a maximum of one hour, we had planned to discuss 10 pictures with each participant. The interviewer therefore asked her to choose a maximum of 10 photographs to discuss. This participant had also gathered photographs from Google.

After discussing the results of the test interviews with the research team, we agreed to add some instructions to the briefing of participants to increase clarity. We decided to add the following instructions to the briefing: ‘You [the participant] can take photographs both with or without yourself in them’, ‘You [the participant] can take photographs yourself or gather them online’ and ‘You [the participant] may choose 10 photographs to discuss during the interview’.

### Participant recruitment and information before the start of the study

We aimed to recruit individuals older than 60 years of age with a low SES, and therefore, we concentrated the recruitment activities in communities characterised by low SES, a contextual measure of SES (Shaver 2007). We used multiple strategies to recruit participants. First, we recruited participants in the local community centre of a low-SES neighbourhood, where a social worker organised group meetings for local older adults. The social worker distributed flyers announcing the study during group meetings. Two researchers (FP and JV) and two research assistants visited a total of four group meetings to provide information about the study. During the meetings, group members received a short presentation about the study, its purpose and its aims. After the presentation, the researchers (FP and JV) and research assistants spoke to each older adult individually to explain the study in detail. Taking or gathering photographs on smartphones was also practiced with potential participants and questions of participants were answered. For potential participants who did not have access to a smartphone, the social worker offered to help take the photographs. Older adults who indicated to be willing to participate also received information in writing about the study and its purpose, along with information about how to take a photograph on a smartphone, informed consent and contact details of the researcher (FP). Thereafter, the researchers and research assistants made individual appointments with the older adults willing to participate to conduct the interviews, which were scheduled two weeks later. After the presentation, some older adults in the group stated that they did not wish to participate in the study, with the most common reasons being deafness, forgetfulness, language barriers or chronic mental and physical conditions. In all, 12 older adults were recruited through this strategy. Two needed assistance from the social worker with taking photographs. One requested assistance from his social support advisor.

Due to the COVID-19 pandemic and the subsequent national lockdown, the researchers and research assistants were no longer able to visit the group meetings in the community centre. The researchers therefore decided to recruit additional participants from within their personal networks as a second strategy. These potential participants received the same verbal and written information as explained above and, two weeks later, interviews were conducted in the participants’ homes or in a local community centre. This strategy yielded another six participants.

**Fig. 1:**
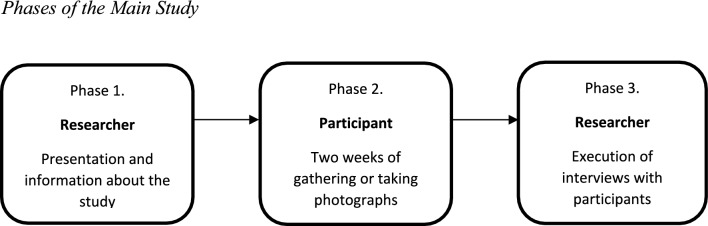
Phases of the main study

Although data saturation was achieved, which means that no new themes were found, after recruitment through the first two strategies, we decided to recruit more participants after the national lockdown to increase the reliability of our study. As a third strategy, we visited a retirement home in a low-SES community, where social workers were leading a group discussion about liveability in the retirement home at that time. All older adults in that group received the same presentation and verbal and written information about the interview as described above. This strategy yielded a total of five participants. After recruiting and informing participants, participants had time to gather photographs during a period of two weeks, and thereafter, participants were interviewed in their homes (see Fig. 1).

### Interview format

The topic list for the interview consisted of three main questions aimed at gaining more insight into the participants’ reflections on positive ageing. The questions were designed based on examples of questions used in other participant-driven photo-elicitation studies (Baker and Wang 2006; Killion and Wang 2000; Novek et al. 2012). When discussing a photograph, the interviewer could ask the following three questions:

- Can you tell me something about this photograph?
- Why have you taken or gathered this photograph?
- What does this photograph have to do with positive ageing?

Depending on the participant’s degree of openness and answers, some additional questions were asked, such as:

- What kind of feeling do you get from this photograph?
- What does this photograph mean to you?
- What does this photograph have to do with health?
- What does this photograph have to do with ageing?

After discussing the photographs, participants were asked whether they had any other ideas about positive ageing that were not represented in the photographs. They were also asked whether the process of taking photographs about positive ageing had changed their thinking about positive ageing or if it had made them more aware about positive ageing.

The part of the interview about positive ageing was followed by questions about the participants’ demographic background and financial situation. These questions were asked in a sensitive manner, as it is known that participants are often reluctant to answer such personal questions (Tourangeau and Yan 2007). To this end, we asked participants whether they were able to make ends meet at the end of the month and whether they needed to save for bigger expenses. At the end of the interview, an evaluation of the method followed. Participants were asked how they had experienced the process of gathering the photographs, as well as about the interview and the use of photographs in an interview, as compared to questionnaires or classic interviews.

### Participants

Data were collected from October 2021 till March 2022. In all, 23 Dutch older adults agreed to participate in the study. The data from two participants were not used for the data analysis, because it came to light that they were in a vulnerable mental state. One participant was a war refugee, and the other participant was experiencing psychiatric problems. During the interviews, these participants were unable to reflect on positive ageing, and they persisted in talking about traumatic war experiences or their mental health condition during the discussion about the photographs. We therefore decided to conduct the analysis with a total sample of 21 Dutch participants living in low-SES communities. Seventeen participants had an individual low SES based on a combination of education, former occupation and financial situation. The mean age of the participants was 75 years old, the majority were female, and participants had a Dutch, German or Indonesian background (see Table 1). All participants were able to read, write, speak and understand Dutch.

**Table 1:** Characteristics of participants

	Participants (*N* = 21)	
	Mean	SD
Age (years)	75	7
	%	*n*
*Gender*		
Female	80	17
Male	20	4
*Marital status*		
Married	33	7
Widow/widower	29	6
Alone	38	8
*Financial situation*		
I make ends meet easily	47	10
I exactly make ends meet and do not require much	33	7
I do not make ends meet easily and need to watch my finances	20	4
*Education*		
None	4.8	1
Primary school	9.5	2
General secondary education	57.1	12
University/higher education	23.8	5
Unknown	4.8	1
*Field of former occupation*		
Healthcare	19.0	4
Transport	9.5	2
Homecare	9.5	2
Retail	19.0	4
Administration	14.3	3
Technic	4.8	1
Chemical laboratory	9.5	2
Education	4.8	1
Recreation	4.8	1
None	4.8	1
*Country of origin*		
Netherlands	71	15
Germany	5	1
Indonesia	24	5

*N* sample size, *SD* standard deviation, % percentage

### Interview procedure

The interview started with the interviewer and the participant introducing themselves, after which participants were asked for consent to making an audio-recording of the interview. All participants gave written consent to use their data and photographs in the study. Although participants were aware that they could stop the interview at any time, none of the interviews was terminated prematurely. The interviews were held in the local community centre (*n* = 12) or in participants’ homes (*n* = 9). Three participants requested the presence of an external professional during the interview. Two participants requested the social worker to be present during the interview. Furthermore, one participant requested his social support advisor of the local government to be present. One married couple requested to be interviewed at the same time. The answers provided by the two spouses were analysed separately. The mean duration of the interviews was 42 min, ranging from 22 to 86 min. The duration depended on the number of photographs and the degree of openness of the participants.

### Data analysis

All interviews were in Dutch and transcribed verbatim by Dutch researchers. Names and residences of the participants were substituted with functional codes to ensure the participants’ confidentiality. There are several ways to define a deductive and inductive approach for the analysis. For instance, inductive can be defined as a bottom-up approach, starting from the experiences of participants (Skjott et al. 2019). A deductive approach can be defined as top-down, in order to test a theory (Fereday and Muir-Cochrane 2006). In this study, we use an inductive approach to stay close to perspectives of the participants. Analysis started with an open-coding phase, during which text fragments were segmented into codes using line-by-line coding. The codes were labelled with phrases or terms used by the participants themselves, thus remaining close to the data. After this open-coding phase, codes with the same meaning were merged together, following discussion with the research team. After the merging process, the 311 codes generated during the open-coding phase were reduced to 93 codes. These codes were divided into 20 categories at a higher level of abstraction. Thereafter, themes were developed along patterns found in these categories, ultimately resulting in 10 themes. During this process, one researcher (FP) and two research assistants analysed the data to ensure the reliability of the findings. All data were analysed using ATLAS.ti Scientific Software Development GmbH version 9.

## Results

In all, 21 participants gathered 194 photographs. Each participant displayed a minimum of four photographs and a maximum of 10 photographs (mean 9). Photographs were collected with smartphones, tablets, online search engines (e.g. Google) or from photo-books and magazines. Two participants had received help from the social worker, and one participant had received assistance from his social support advisor when gathering the photographs. The number of photographs and the themes displayed on the photographs differed for every participant. Six participants had gathered more than 10 photographs. These participants were asked to select 10 photographs that would be discussed during the interview. Photographs often displayed a combination of themes (e.g. eating with children and grandchildren). In addition, most participants gathered multiple photographs displaying the same theme (e.g. a collection of vacation photographs). We categorised the photographs according to the theme discussed during the interview. A schematic overview of the frequency of specific themes displayed on the photographs is presented in Table 2.

**Table 2:** Overview of participants’ photographs

Theme	Number of photographs (total 194)
*Social health*	
Children and grandchildren (family)	44
Partner	3
*Activities*	
Creative hobbies (painting, writing, knitting, public speaking, playing music)	24
Vacation	21
Other activities (dancing, playing (online) games, watching series/movies)	10
*Nature and animals*	
Pets and animals	16
Flowers	11
Nature	8
*Food and drinks*	
Food	24
*Home*	
Home and home decorations	16
*Participant self*	
Participant self	6
Photographs of participants’ youth	4
*Other*	
Clothes	2
Work	2
Religion	1
Money	1
Quote about ageing	1

In this section, we discuss the results of the interviews according to the themes that were discussed most often and those that participants connected with positive ageing during the interviews (see Table 2). Not all photographs or themes discussed during the interviews were connected with positive ageing. For instance, participants also talked about and photographed home decorations or showed photographs from their youth, but they did not necessarily draw any connections between these themes and positive ageing. The themes that were discussed most often were Social Health, Activities, Nature and Animals, and Foods and Drinks. The themes Mental Health and Perspective on Ageing were also discussed frequently during the interviews, although they were not photographed.

### Social health and positive ageing

Almost all participants mentioned that aspects of social health were important to positive ageing. In particular, participants mentioned close relatives (e.g. partner, children, grandchildren) as a source of considerable joy, fulfilment in life, love and happiness. Other social contacts (e.g. neighbours, friends) were not mentioned often. Participants mentioned that doing activities with grandchildren (e.g. playing, walking, babysitting) made them feel young again, gave them a purpose in life and much joy. Some participants explained that, when their children and grandchildren are healthy, they feel healthy as well. Most participants talked with pride about their children and grandchildren.

*If we talk about positive ageing, they are my everything. I live for them.—P15*

Only one participant with children did not associate children with positive ageing.

*Interviewer: Do your children have anything to do with positive ageing?*

*P19: No why? When I do not see them for two weeks, it does not influence me at all.*

Participants mentioned their spouses or partners much less often than they mentioned their children and grandchildren. Those who did mention their partners spoke of their happiness in being together or about the physical health or loss of the partner. Poor physical health of a partner had a particularly strong effect on the lives of participants (e.g. due to the care they needed, the activities that participants were no longer able to do, the positive mindset required to support the partner).

### Activities and positive ageing

Participants often showed photographs of activities (e.g. creative hobbies, vacations). In most cases, these activities were done together with other people. Examples included painting with other older adults in a group or going on vacation with family. Participants did not necessarily draw a connection with positive ageing when describing these activities. They often mentioned simply enjoying time spent with other people. The activities were mentioned as a strategy for remaining positive in life when feeling down (e.g. by going to paint).

*If I feel down and I start painting, I always choose the colour yellow, because yellow makes me happy. That is just the way I am.—P9*

### Nature and animals and positive ageing

For most participants, nature was a place to take rest, and it contributed to a peaceful state of mind. Most participants enjoyed spending time in nature or, when less mobile, looking at photographs or paintings showing nature sceneries. For the participants who were less mobile, flowers at home (e.g. in the garden or on a balcony) also contributed to feelings of happiness.

For most participants, having a dog or cat was also an aspect of positive ageing. Most pet owners mentioned the love they received from their pets as major contributor to positive ageing. Some participants compared the love they felt for their pets to the love they felt for their children. In addition, it forces some participants to go for a daily walk and have some contact with other people who have pets.

*We love our animals; they make us really happy.—P11*

### Food and drinks and positive ageing

Participants often gathered photographs depicting food or drinks. Most participants talked about eating with other people (e.g. family, other older adults) as something that made them feel happy and connected. There was a difference in this regard between participants of Dutch origin and those of Indonesian origin. Indonesian participants talked about eating together more often, and they explained that making other people happy with their food gave them a feeling of fulfilment. These participants also identified the importance of eating together with family as part of their upbringing and culture.

*For me, it is happiness; it gives me much joy. I was raised with food and cooking.—P3*

### Mental health and positive ageing

Although most participants did not mention mental health specifically when discussing the photographs, they often mentioned aspects of mental health that had contributed to their positive ageing. Examples included staying positive, enjoying life and happiness. One participant explained that enjoying life and feeling happy is the same as being healthy, despite her physical problems.

*My health is not that good, but I feel happy. And if you are happy, you are healthy right?—P11*

Another aspect that most participants mentioned often is the acceptance of new situations when growing older (e.g. declining health, loss of loved ones). All participants mentioned simply keeping going on with life as a strategy.

*You will have to deal with it. Simple. You cannot complain. This is what the new situation is, now is now, you should make something of it. It is quite simple.—P10*

One participant found it very difficult to find any positive aspects of ageing. This participant defined ageing as losing her physical health, home and people around her. She was not yet able to accept her new situation.

### How to age positively

When specifically asked ‘What is positive ageing?’, participants gave a variety of answers, although most had the same core aspects. Most participants equated positive ageing with having a positive mindset, looking at the bright side of life, giving love to other people, trying to participate in the society and enjoying the little things in life.

*We all become older, but you can choose: will you stay inside, sitting around waiting to die* [Dutch: achter de geraniums zitten]*? I think you get worse very fast then. I could still move mountains. I really want to.—P5.*

In addition to talking about the positive aspects of ageing, some participants also mentioned the difficulties associated with ageing. Participants mentioned having difficulty accepting their physical disabilities and limited mobility. Some participants also mentioned having trouble accepting changes in their appearance due to ageing (e.g. greater difficulty losing weight, wrinkles, hair loss). They also noted that everything seemed to be moving faster while they felt as if they were moving more slowly. For example, most participants had trouble keeping up with digital media (e.g. telephones, laptops, internet).

*Everything goes slowly. Before, I could clean all the windows in one hour. At this moment, I need half the afternoon. I just need help sometimes, and that is not positive at all.—P19.*

### Evaluation of the photo-elicitation method

Participants were asked whether their participation in the study had changed their thinking about positive ageing and that it had made them more aware of positive ageing. Their answers varied. Some participants said that they had not become more aware, and others reported that they had talked about the study with children, grandchildren or other older adults after the presentation, and that this had increased their awareness of positive ageing. This was particularly true for the participants who were living in a retirement home.

*Immediately after the presentation, I kept thinking, what is positive about ageing?—P19*

All participants were asked how they had experienced the process of gathering the photographs and how they had experienced the interview. All participants were positive about the method and said that they had found the interview enjoyable, relaxing, interesting and pleasant. Compared to classical interviews or questionnaires, most participants considered the photo-elicitation method more enjoyable. Participants were especially appreciative of the personal contact with the interviewer and some participants explained that they had also learned from the experience. By using photographs, participants reported that it had been easier to talk about positive ageing, as the photographs had served as a guide during the interview. Two participants explained that the photographs had enhanced the reliability of their statements.

*You can see that we are not just saying something; it is real.—P21*

Participants used different methods to gather photographs, mostly depending on their digital abilities and the assistance they received from within their networks. Some participants had difficulty using smartphones or tablets and had instead gathered the photographs with help from others (e.g. social workers, children or grandchildren) or used magazines or photo-books.

### Participants’ understanding of the photo-elicitation method

The two participants who had received help from a social worker and one participant who had received support from his social support advisor mentioned having had trouble understanding the photo-elicitation method and questions. These participants had asked the social worker or social support advisor multiple times for input and support during the interview, and we noticed that they seemed to depend on the perspective and opinion of the professional. This affected the interview and the data. We did not experience any other misunderstandings with the other participants.

*Interviewer: What does this photograph have to do with positive ageing?*

*P2: What do you think?* [directed towards the professional].

*Interviewer: I would like to hear your* [the participant’s]* perspective.*

*P2: [long silence]*

## Discussion

The primary aim of this study was to explore the perceptions of older adults with low SES concerning positive ageing, as well as their experiences with the photo-voice method. The results indicate that the most common perceptions of positive ageing involved spending time with loved ones (especially children and grandchildren), having a positive mindset in life and, for participants from Indonesian origin, eating together with family and friends. Activities that were frequently mentioned as contributing to positive ageing included being creative, going into nature and spending time with pets. Although the participants’ answers concerning the meaning of positive ageing varied, they shared the elements of accepting becoming older, maintaining a positive mindset and enjoying the little things in life.

Our results confirm those reported in previous studies, which highlight the importance of social contacts for the health and well-being of older adults (Douma et al. 2017; Rafnsson et al. 2015; Ten Bruggencate et al. 2018; Umberson and Montez 2010) in addition to SES (Pinquart and Sörensen 2000). Participating in leisure activities with other people is known to satisfy the social needs of older adults (Ten Bruggencate et al. 2018). This was confirmed by our participants, who often photographed other people and discussed activities with other people during the interviews. Eating together with family and friends was photographed and mentioned often, especially by participants of Indonesian background. This might have been because eating together was identified as an important aspect of the Asian food culture, and it is known to be a facilitator for social interactions and psychological well-being (Connor and Armitage 2002). In a qualitative study, Tkatch et al. (2017) report that older adults defined health in terms of psychological and social components, as well as with reference to the need for resilience and coping skills to deal with health-related (or other) challenges. These observations are in line with the results of our study, and they highlight the importance of social ties and resilience for positive ageing in older adults with a low SES.

Participant-driven photo-elicitation seems to be a suitable method to use in research with this target group of older adults with a low SES. All participants enjoyed the method, and the engagement of participants seemed to be higher than it had been in previous interviews without participant-driven photo-elicitation (Castleden et al. 2008; Platzer et al. 2021a; 2021b). For instance, we noticed that participants had talked with other older adults or family members about the study, and that the use of photographs had made it easier for them to talk during the interview, as they had served as a guide. These results are in line with other studies on participant-driven photo-elicitation, and they emphasise the potential of this method for gerontological research (Mysyuk, and Huisman 2020; Pain 2012).

Although no participant explicitly admitted to having difficulty gathering photographs, we noticed that those with limited digital skills seemed to have trouble using electronic devices to gather photographs. Some participants received help from their children or grandchildren, or they used magazines to gather photographs. These findings are in line with research by Novek and colleagues, who suggest that some older adults might need support when gathering photographs (Novek et al. 2012). At the end of each interview, we asked each participant whether there were any aspects of positive ageing that they had not been able to gather photographs on. Some participants mentioned having trouble gathering photographs of abstract subjects (e.g. happiness). It could thus be the case that non-tangible aspects were under-represented in the photographs taken for this study. Most participants nevertheless stated that they had been able to gather all the photographs they wanted.

Our study has several important strengths. First, after reaching data saturation during the coding phase, we decided to interview five more participants to improve the reliability of the study (Rose and Johnson 2020). In addition, throughout the interviews and the coding and analysis of the data, a team of researchers and research assistants worked closely together to improve the validity of the results (Campbell et al. 2013).

Despite its strengths, the study was also subject to several limitations that should be mentioned. First, two participants received assistance from a social worker with gathering the photographs, and another received support from a social support advisor. These participants seemed to rely on the opinions of the professionals, and this might have affected the data. For future research, we recommend pretesting participants by holding a short interview with potential participants before the start of the study, in order to explore whether they are able to participate independently, to avoid any potential influence from an assisting individual.

Second, one could argue that some topics important for positive ageing of participants could not be photographed, because they were abstract concepts, such as organising a future event, or because someone did not want to be photographed, such as a partner. However, we tried to prevent this by explicitly asking participants whether they had any other ideas about positive ageing that were not represented in the photographs.

Third, although we recruited all participants in neighbourhoods with low SES, a contextual SES measure (Shavers 2007), it was difficult to determine whether participants had a low individual SES. We asked about education and former occupation, but not about income, as this is a sensitive subject. As an alternative, we asked about their financial situation, whether they were able to make ends meet, but this didn’t seem to be a good distinguishing question as an indication of wealth, especially when participants did not want to elaborate on their answer. Overall, it is challenging to determine SES in older individuals because indicators are age- and gender-dependent and susceptible to errors or non-responses (Shavers 2007). For future research aiming to include only participants with individual low SES, a brief intake interview prior to the start of the study may help to select only older adults with low education or occupation.

Fourth, we found that eating together with family and friends was photographed and mentioned often, especially by participants with an Indonesian background. Although these findings suggest that the country of origin had an influence on perceptions concerning positive ageing, more participants from a larger variety of ethnic groups are needed in order to draw any conclusions about cultural influences on experiences of positive ageing among older adults with a low SES.

## Conclusion

This photo-voice study among older adults living in low-SES neighbourhoods revealed that perceptions concerning positive ageing involved a combination of spending time with loved ones, activities, nature, pets, and depending on country of origin eating together. Additionally, accepting losses and enjoying life was perceived as important for positive ageing. An integrated approach could be directed at these perceptions in positive health programmes for older adults with a low SES. As all participants enjoyed the method, and it facilitated the conversion, photo-voice can be a useful tool for further exploration of positive ageing among older adults with low SES with various backgrounds.

## Competing interests

The authors declare no competing interests.

## Data Availability

No datasets were generated or analysed during the current study.
